# Decreased CD10 Expression in the Bone Marrow Neutrophils of HIV Positive Patients

**DOI:** 10.4084/MJHID.2010.032

**Published:** 2010-11-15

**Authors:** Annemarie van de Vyver, Eluned Delport, Adele Visser

**Affiliations:** 1 Department Internal Medicine, Kalafong Hospital, University of Pretoria; 2 Department Internal Medicine, Steve Biko Academic Hospital, University of Pretoria; 3 Department Clinical Pathology, University of Pretoria, National, Health Laboratory Services

## Abstract

**Background:**

HIV-1 infection is associated with various quantitative and qualitative changes in haemopoietic cells. Clear distinction between primary myelodysplastic syndrome (MDS) and secondary dysplasia may not always be possible. Adjunctive analyses used in the diagnosis of MDS include cytogenetics and flow cytometry (FCM). Much focus has been placed on establishing FCM guidelines aiding in the diagnosis of MDS, and to distinguish this condition from secondary dysplastic changes. One of the parameters often cited is the CD10 expression on the granulocyte population, as this marker denotes granulocytic maturation.

**Aims:**

To determine the expression level of CD10 on granulocytes in HIV positive patients.

**Methods:**

In total, 117 HIV-1 positive and 29 HIV-1 negative patients were included in this study. Bone marrow aspirate samples were evaluated in terms of morphological abnormality as well as CD10 expression on the granulocytic population.

**Results:**

The average CD10 expression among the HIV-1 positive patients were markedly reduced, at 18.4%, and 113 patients (96.6%) of these patients had expression levels below 50%.

**Discussion:**

Disease conditions causing secondary dysplasia, especially HIV-1 infection, is associated with a marked reduction in CD10 expression on the granulocyte population independent from the presence of myelodysplastic features. This marker is therefore of doubtful significance as a diagnostic tool in distinguishing between primary and secondary dysplasia.

## Introduction:

Infection with the Human Immunodeficiency Virus (HIV-1) is frequently associated with haematological abnormalities,^1^,^2^ including cytopaenias^3^ and dysplastic features.^4^,^5^ The aetiology for these changes are often multifactorial^6^ including features of peripheral destruction,^3^ bone marrow suppression due to drugs, 7 opportunistic infection^8^ or direct effect of HIV-1 on bone marrow precursor cells.^9^,^10^ HIV-1 infection has also been directly associated with myelosuppression with various possible underlying mechanisms postulated, including direct infection of haemopoietic progenitor cells,^26^,^1^ stromal cell functional impairment, toxic effects of HIV-1 proteins and alterations in the local cytokine milieu.^1^ The presence of dysplastic haemopoiesis in the bone marrows of HIV-1 positive patients have been well described,^23^ however, reports on MDS in HIV-1 positive patients are rare.^24^,^23^ Superficially these dysplastic changes can be morphologically similar to the dysplasia seen in primary myelodysplasia.^30^

The diagnosis of MDS calls for multidisciplinary evaluation of morphological, haematological and cytogenetic findings.^4^ As the more sophisticated investigations like cytogenetics yields aberrant findings in only about 20 to 70% of cases,^11^,^12^ novel methods of diagnosis are constantly evaluated. Recently, more emphasis has been placed the use of flow cytometry (FCM) in the diagnostic work-up of patients with MDS.^27^,^20^ Decreased or absent CD10 expression in the granulocyte series has been associated with MDS, and has been cited to occur in as much as 45% of MDS cases.^16^ Use of this marker has been proposed as a criterion in either diagnostic scoring systems^4^ or as supportive diagnostic criteria.^27^,^16^

However, expression levels of CD10 have not been evaluated in various other conditions, including known causes of secondary dysplasia, particularly HIV-1 infection.^15^ A recent publication from the European LeukemiaNet Working Conference^16^ re-emphasized use of various parameters in the evaluation of patients with MDS, in an attempt to establish consensus guidelines in the diagnosis of MDS. For this reason, it remains imperative to fully understand the effect of various conditions on CD10 expression on the granulocyte population, as this may impact on future diagnostic criteria.

This is the first study to the authors’ knowledge to investigate CD10 expression in granulocytic cells on bone marrow aspirate samples from HIV-1 positive patients with non-neoplastic haematological disorders.

## Materials and Methods:

### Patient Population:

In total, 117 HIV-1 positive and 29 HIV-1 negative patients were included in this study. None of the HIV-1 positive patients had been initiated on highly active antiretroviral therapy (HAART) at the time of investigation and were in varying stages of HIV-1 disease, as reflected by CD4 counts. The presence of concurrent opportunistic infections was not known. Exclusion criteria included 1) patients younger than 12 years of age, 2) a diagnosis of any haematological neoplasm and 3) HIV-1 negative test (in the negative control group) performed more than 12 months from bone marrow examination. Flow cytometric data as performed on bone marrow aspirates were collected on all cases. None of the HIV-1 negative control group was considered to have primary myelodysplasia based on morphology or karyotyping.

### Bone Marrow Morphology:

Of the 117 samples included, only 70 included sufficient smear samples for evaluation of morphological features. The iron stain was used to evaluate the presence of sideroblasts, and quantified as low (less than 20%), normal (20 to 30%) or increased (more than 30%), as well as the presence of pathologically overloaded forms.

### Flow Cytometric Analysis:

All bone marrow specimens were collected in ethylene-diaminetetraacetic acid (EDTA) or sodium-heparin tubes and processed within 24 hours. The white blood cell (WBC) count was determined using FLOW-COUNT, Beckman-Coulter (Miami, USA) and corrected to within the reference range (4–10 ×10^9^/L) by either dilution with RPMI or buffy coat harvesting by density gradient extraction. A total volume of 100 μl of the bone marrow specimen was mixed with 2 ml of ammonium chloride to lyse red blood cells. The specimen was incubated for 10 minutes and centrifuged for 10 minute at 2000rpm. Viability was subsequently determined by using propidium iodide. Only specimens with a value above 75% viability were used.

**Figure 1:**
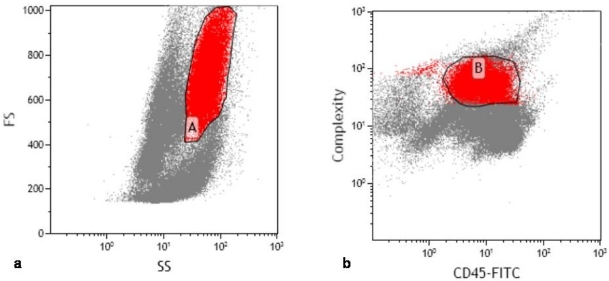
Granulocytes defined based on a. Side scatter (SS) versus Forward Scatter (FS) (population A) and b. CD45 versus SS (population B).

**Figure 2:**
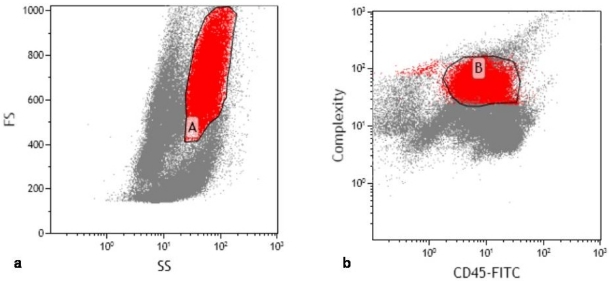
CD10 expression evaluated by a. CD5 FITC versus CD10-PE and b. CD10-PE versus CD19-ECD. Both sets were gated on population A as defined in figure 1, identified as granulocytes. Two discrete populations can be demonstrated in each defined as either CD10 positive (population C and I) or negative (populations D and H).

According to our laboratory’s standard operating procedure, 10 μl of CD10 monoclonal antibody was added to 100 μl of the bone marrow. Phycoerythrin (PE)-CD10 by Beckman-Coulter (Miami, USA) was used. Three colour flow was performed with the Cytomics FC500 flow cytometer or XL-MCL, Beckman-Coulter (Miami, USA) equipped with a 15-mW argon laser (excitation at 488mm). Antibody panel used included CD5-Fluorescein isothiocyanate (FITC) vs CD10-PE vs CD19-PE-Texas red (ECD). Granulocytes were defined using side scatter versus forward scatter (Figure 1a) and CD45 (dim) versus side scatter (Figure 1b). CD10 expression was determined within this population. Compensation within this gate was performed by also incubating cells with appropriate fluorochrome-coupled isotype control antibodies.The CD10 expression was quantified from the CD5-FITC versus CD10-PE analysis (Figure 2a) and verified by comparison to CD10-PE versus CD19-ECD (Figure 2b). CD10 PE was used to estimate the total CD10 expression of the granulocyte population. Standardization of instrument electronics was done using Flow Check Fluorospheres calibration beads by Beckman-Coulter (Miami, USA) according to the manufacturer’s recommendations. Compensation was adjusted using FITC-CD4/PE-CD8/PerCP-CD3-stained cells. Isotypic controls were used as negative controls to eliminate non-specific binding. In each cell preparation, 15 to 30 thousand total events were collected. Data were analyzed with CXP cytometer software, Beckman-Coulter (Miami, USA) gating the total leukocyte population.

## Results:

### Patient population:

Ages for the patients varied from 23 to 77 years (mean age of 37 years) for the HIV-1 positive patients and 12 to 85 years (mean age of 42 years) for the HIV-1 negative patient group (Table 1). Although not HIV-1 infected, the latter group had various underlying medical conditions which is associated with secondary dyplasia. This included 7 cases with haematinic deficiencies, be it iron, folate, vitamin B12 or a combination of these. Also included were 4 patients with systemic sepsis, 4 with auto-immune disease, 3 each with chronic renal failure and chronic inflammatory diseases, 2 with thyroid disease and 1 with liver cirrhosis. Three of the patients were admitted with haematological disorders involving either platelets or red cells.

### Bone Marrow Morphology:

Various dysplastic features could be noted in all cell lines (Table 2). Of note was the presence of trilineage dysplasia in one third of the patients, and bilineage dysplasia in another third. Dysplasia was noted in only one lineage in 26% of cases and no dysplastic features were noted in 9%. Blast counts were not increased in any of the HIV-1 positive patients, and monocytosis was a rare finding. Although dysplasia was a frequent finding, it could not be demonstrated in more than 10% of the myeloid lineage, and therefore did not meet the WHO diagnostic criteria for primary myelodysplastic syndrome.

### Flow Cytometric Analysis:

**Figure 3.**
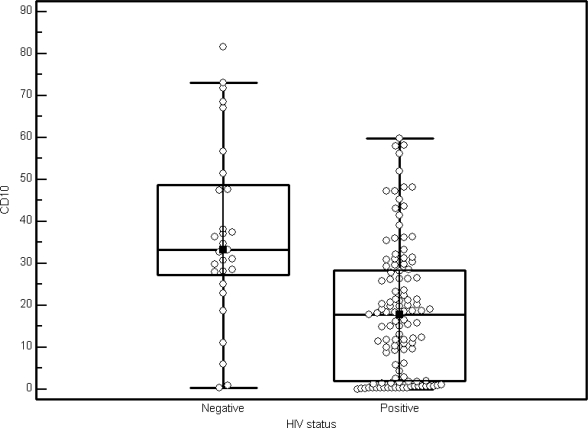
CD10 expression depicted as a Box and Whisker plot

In the HIV-1 positive study group, the average CD10 expression on granulocytes were 18.4% (SD of 15.83) compared to the HIV-1 negative group with levels of 37.1% (SD 21.2) (Figure 3). In the HIV-1 positive group 113/117 (96.6%) patients had a total CD10 expression of less than 50%, in contrast to 22/29 (75.9%) the HIV-1 negative group (table 1)(p<0.0001). A left shift in the granulocyte lineage could be demonstrated in <30% of cases. Exclusion of these left-shifted cases did not have a statistically significant impact on the data, increasing the average CD10 expression to 18.5%. No correlation between CD10 granulocyte percentage and CD4 counts (as a marker for HIV-1 disease progression) could be demonstrated in the HIV-1 positive population (p>0.34).

## Discussion:

Changes in bone marrow morphology associated with HIV-1 infection has been evaluated by various authors.^1^,^5^,^8^,^28^ These studies showed that certain findings are common to both HIV-1 and MDS. These include increased bone marrow cellularity, left shift in granulocytes, presence of giant band cells and megaloblastoid changes in the erythroid lineage, consistent with the findings in the current study. Macrocytosis can also be present especially secondary to drugs administered in the treatment of HIV-1. Findings found in primary MDS, in contrast to HIV-1 dysplasia include an increase in blast cells, hypogranularity as well as hyper- or hyposegmentation of granulocytes and micromegakaryocytes with atypical lobulation,^23^ but the blast counts were not elevated. These finding were not in keeping with the current study, as hypogranularity, hyposegmentation, hypersegmentation and the dysplastic features in megakaryocytes were relatively common among our HIV-1 positive population. These findings have been cited as useful in delineating between these two conditions, however, changes may be subtle. Depending on the different subclasses of primary MDS, degrees of dysplasia may also vary.^23^ Therefore, distinguishing morphologically between primary and secondary dysplasia can be very troublesome.

For all these reasons, establishing the underlying cause of dysplasia should be approached as a multidisciplinary work-up. Although considered diagnostic, cytogenetic analysis typically contributes to only 40% of cases, showing a variety of abnormalties.^4^ Consequently, scoring systems have been advocated for use in diagnosis to improve sensitivity and specificity of diagnosis.^4^,^16^ Various flow cytometric parameters have been evaluated as useful diagnostic indicators in MDS, including use of CD10 expression on granulocytic cells.^21^

Expression of CD10 has been examined as a marker of granulocyte maturation in various studies,^12^,^14^ as it is expressed by terminally differentiated neutrophils.^25^ Levels have been evaluated by numerous authors, showing reduced levels on granulocytes varying from 20 to 70%,^11^ depending on the subtype of MDS. Secondary dysplastic changes can occur due to various conditions and should be excluded before the diagnosis of MDS can be considered.^3^ With specific relevance to HIV-1 positive patients, long term use of zidovudine (AZT) has been shown to induce dysplasia in mouse studies.^2^ However use of gancyclovir and trimethoprim-sulfamethoxaxole (standard prophylactic antibiotic used according to the South African guidelines) have not been shown to induce dysplasia in vivo.^23^ The HIV-1 negative group also included some patients with other causes of secondary dysplasia.^31^ This again correlates with the finding of reduced CD10 expression in this population as well, where almost 76% of patients had levels below 50%. In light of these findings, decreased CD10 expression may correlate with myeloid dysplasia, not only in primary, but also secondary dysplastic conditions.

Decreased CD10 expression has been associated with increased susceptibility to infections, likely due to its role in neutrophil chemotaxis and coordination of the inflammatory response.^22^ This becomes clinically evident amongst neonates, patients with burns and those with systemic sepsis, all of which have been shown to have both reduced CD10 expression on their granulocytes, and a degree of immunosuppression.^6^ The clinical implications of the reduced CD10 expression noted in HIV-1 positive patients remain unknown.

The diagnostic utility of FCM has not been evaluated in HIV positive patient’s bone marrow samples. The interrelationship between the presence of dysplasia and the effect on the function of the bone marrow has also not been studied. In this study, HIV-1 positive patients showed a significant reduction in CD10 expression, with 96.6% of the patients showing expression below 50%. This calls into doubt the use of this marker as an adjunct in the diagnosis of MDS.

## Conclusion:

Flow cytometry is a useful adjunct in the diagnosis of MDS. Although a single antigen abnormality cannot be used in isolation to diagnose MDS, it remains of paramount importance to evaluate the merit of each antigen within clinical context. Utility of specific markers should however be carefully evaluated as causes of secondary dysplasia may also elicit similar immunophenotypic abnormalities, currently advocated as markers specific for primary myelodysplasia. A full understanding needs to be gained on immunophenotypic changes associated with various stages of HIV-1 infection, to so facilitate use of this diagnostic adjunct in this patient population. The impact of these aberrancies needs to be clinically correlated with the degree of immunological dysfunction.

## Figures and Tables

**Table 1.** Baseline demographic and results of study population

**Variable**	Study population *n=148*	
	**HIV-1 positive patients***n=117*	**HIV-1 negative patients** n=29
**Age (in years)**	37 (23–77)	42 (12–85)
**Average CD4 count**	129±151	N/A
Average CD10% expression*	18.40%	37.10%
Number of subjects with CD10% expression* <50%	133 (96.6%)	22 (75.9%)

* CD10% expression on granulocyte population

**Table 2.** Dysplastic features in HIV-1 infected patients.

	**Dysplastic Features** n=70	**Absolute** (percentage)
**Bone marrow cellularity**	Hypercellular	31 (44%)
	Normocellular	35 (50%)
	Hypocellular	5 (6%)

**Granulocytic Lineage**	Left shift	21 (30%)
	Giant Metamyelocytes	33 (47%)
	Monocytosis	1 (1%)
	Hypogranularity	3 (4%)
	Hyposegmentation*	24 (34%)
	Hypersegmentation	9 (13%)

**Erythroid lineage**	Red cell dysplasia§	57 (81%)
	Macronormoblasts	11 (16%)
	Megaloblasts	25 (36%)
	Multinuclear erythroid cells	14 (20%)

**Megakaryocytic lineage**	Micromegakaryocytes	4 (6%)
	Atycal nuclear configurations	26 (37%)

**Sideroblasts (n=46)**	Decreased	35 (22%)
	Normal	1 (2%)
	Incresed/pathologically overloaded	10 (76%)

* Hyposegmentation includes Pseudo-Pelger-Huet forms and ring forms

§ Red cell Dysplasia includes uneven haemoglobinization, ragged cytoplasm and intercytoplasmic bridging
